# Reduction in Chronic Disease Risk and Burden in a 70-Individual Cohort Through Modification of Health Behaviors

**DOI:** 10.7759/cureus.10039

**Published:** 2020-08-26

**Authors:** Thomas J Lewis, Jason H Huang, Clement Trempe

**Affiliations:** 1 Chronic Disease, Massachusetts Institute of Technology, Knoxville, USA; 2 Neurosurgery, Baylor Scott & White Medical Center - Temple, Temple, USA; 3 Ophthalmology, Beth Israel Deaconess Medical Center, Harvard Medical School, Boston, USA

**Keywords:** chronic disease management, population health, health literacy, health risk score, serum biomarkers, cytokine storm syndrome, prevention, disease reversal, inflammation, health behaviors

## Abstract

Introduction

Health risk factors, including lifestyle risks and health literacy, are known to contribute to the chronic disease epidemic. According to the Centers for Disease Control and Prevention (CDC), chronic diseases account for 90% of healthcare costs, morbidity, and mortality. In the United States, healthcare providers attempt to modulate a limited set of risks. However, chronic diseases continue to proliferate despite expansion of wellness programs and drugs to manage and prevent chronic conditions. Pandemics, exemplified by severe acute respiratory syndrome coronavirus 2 (SARS-CoV-2), show that people in good health suffer mortality rates at 10% the rate compared to those with pre-existing chronic conditions. Healthcare costs and morbidity rates often parallel mortality rates. New root-cause risk and health tools that accommodate low health literacy and are linked to personalized health improvement care plans are needed to reverse the chronic disease epidemic. Reported here is a study on 70 manufacturing employees in the Midwest US using a personalized and group approach to chronic disease reversal and prevention which may also find utility in pandemic severity and policy decisions.

Methods

Health, lifestyle, behavior, and motivation data were collected on 70 individuals at the beginning of a nine-month disease reversal and prevention program. The data were updated every two to six months over the period. Inputs included information from a novel health risk assessment, serum biomarkers specific for chronic disease, and traditional medical information. Using all these data we generated robust, personalized, and modifiable care plans that were implemented by the participant and guided by a care team including health coaches and medical providers. Periodic renewal of profile data and biomarkers facilitated adjustment of care plans to optimize the path toward health goals set mutually by the participant and the care team.

Results

Ninety percent of participants experienced a favorable reduction in chronic disease biomarkers. The reduction in serum biomarkers coincided with a reduction in disease and risk attributes based on medical chart data and before and after interviews. Hemoglobin A1C, for example, lowered in all but one participant concomitant with reported improved energy and reduced need for medications in the majority of participants. Markers of inflammation lowered across the population. Most importantly each individual reported improvement in their overall health.

Conclusions

This simple, inexpensive, root-cause based risk and health approach generates a “do no harm” action plan that guides a care team, including the participant, on a path to improved health. The data demonstrate that changes in a novel risk calculator score coincide with changes in sensitive biomarkers for chronic disease. When the risks of an individual are reduced, the biomarkers reflect that change with self-reported wellbeing also improved. This program and process may be of value to society plagued with escalating levels of chronic disease and merits further study and implementation.

## Introduction

Developed nations, and particularly the United States, continue to confront a chronic disease crisis. The World Health Organization (WHO) reported that in 2010, non-communicable chronic diseases including: cardiovascular diseases, diabetes, cancers, and chronic respiratory diseases, accounted for 2/3 of deaths worldwide [1]. The Institute of Medicine reported that America is less healthy compared to high income nations in obesity, diabetes mellitus, heart disease, chronic lung disease, and disability [2]. The Organization of Economic Cooperation and Development (OECD) tracks the health of 36 developed nations. The U.S. scores in the lower half among these 36 nations on all major indicators of health, and longevity. When considering that the per person per year cost of healthcare in the United States is more than two and a half times that of the OECD nation average, yet our residents live 2.5 years less, a health paradox exists in the United States. This U.S. Paradox is the worst cost-to-value benefit for chronic disease outcomes compared to the average of 35 other developed nations. The chronic disease management system is failing people at both ends of the health spectrum. A root of the problem is that health and prevention recommendations currently supported by the major medical societies have proven ineffective at reversing or preventing chronic diseases. Laboratory tests in common use remain of limited scope and provide little insight into chronic health status. Pharmaceuticals prescribed based on test results have poor absolute statistical success at preventing or reversing disease. These assertions are borne out since 90% of the nation’s nearly $4 trillion in annual health care expenditures are for chronic conditions per the CDC. And the situation is not improving, for example, cardiovascular disease mortality, managed with statin drugs, blood pressure medications, and other usual care approaches across broad members of the U.S. population, increased nationally by 4.3% between 2011 and 2016 [3].

On average, residents of the United States with five or more chronic conditions spend 14 times more on health services than people with no chronic conditions [4]. As of 2014, 60% of U.S. adults had at least one chronic condition, and 42% had more than one chronic condition. Five percent of the population accounts for an estimated 49%-53% of total health care expenses [5]. The 15 most expensive health conditions account for 44% of total health care expenses. The financial and productivity costs impact our corporations, who fund over half of the national healthcare at a price of roughly 4% of their gross revenues. And much of this cost is segmented in high-cost beneficiaries where, for example, the top 1% of claimants cost $150,000/y compared to the population mean of $5800/y. In a report compiled by the Health Care Cost Institute, there is a surprising large turnover from year to year among the highest cost healthcare spenders. Three out of five top spenders in any given year were low or moderate spenders in the prior year. In 2015, only 39% of the top 5% of spenders were in the top 5% of spenders in 2014. Moreover, this trend was consistent in each year from 2009 to 2015. There is a need for better predictive analytics to determine who is and, more importantly, will be in this significantly high-cost segment of any population as a current tool, claims data, lacks predictive power.

In pandemics, standard tests provide little information on projected outcomes, rather they simply indicate exposure and potential immunity. Healthy people are much less likely to die compared to unhealthy or older people. Physiological health, the main concern of practicing clinicians, is not well characterized through these tests. Further, the main cause of death appears to be Cytokine Storm Syndrome which is driven by innate, not adaptive immunity [6]. Thus, antibody testing does not adequately describe disease risk or severity. Validated data on severe respiratory viral diseases and the correlation between mortality, immunocompromised status and existing chronic conditions in infected individuals indicate that a broad set of blood-based biomarkers may best serve to stratify risk and to set policy on containment strategies in populations [7]. Currently, the policy is being established with an incomplete set of evidence. In vivo blood biomarker analysis offers considerable opportunities for individual and population risk measurement. These tests afford fast analytical turn-around time, quantitative measurement, accessibility, serial monitoring and ready availability. In some instances, rapid and continuous monitoring is available.

The measurement of and changes to a broad range of modifiable risk factors, and biomarkers connected to immune system activity through cytokine surrogates, offers the most important opportunity for the prediction of disease and improvement in global chronic and pandemic disease status. Most industries recognize the value of early problem intervention. In the waste management industry, for example, there is a clear hierarchy of:

1. Source reduction, 2. Recycling, 3. Treatment, and 4. Land disposal. In healthcare, there is also a potential for a four-tiered approach to health maintenance: 1. prevention, 2. mitigation of asymptomatic disease in people with elevated predictive biomarkers, 3. mitigation upon the earliest detectable signs of early disease (dry macular degeneration is an example), and 4. advanced root-cause mitigation approaches within disease management approaches. Most of the efforts in today’s healthcare is on disease management with usual care which is only a small part of this suggested four-tiered approach.

The WHO addressed major causes of chronic diseases with modifiable risk factors being: unhealthy diet; physical inactivity; and tobacco use. In addition, the WHO stated “These causes are expressed through the intermediate risk factors of raised blood pressure, raised glucose levels, abnormal blood lipids, overweight and obesity. The major modifiable risk factors, in conjunction with the non-modifiable risk factors of age and heredity, explain the majority of new events of heart disease, stroke, chronic respiratory diseases and some important cancers. The relationship between the major modifiable risk factors and the main chronic diseases is similar in all regions of the world.” Studies show that the U.S. experiences the same risks as exist globally. Dietary factors, alone, are associated with nearly half of all cardiometabolic deaths. The highest proportions of cardiometabolic deaths were estimated to be related to excess sodium intake, insufficient intake of nuts/seeds, high intake of processed meats, and low intake of seafood omega-3 fats. Dramatic changes in disease rates among migrating populations indicate that the primary determinants of these diseases are not genetic but environmental factors, including diet and lifestyle [8]. Studies on twins separated at a young age corroborate that chronic disease is much more related to environmental factors.

Expansion of the depth and breadth of risk assessment and concomitant prevention and disease amelioration programs represent an unmet healthcare need. A well-studied disease prevention arena is corporate wellness programs. Most of these programs rely on “usual care” that includes: basic dietary recommendations, weight loss, smoking, alcohol consumption and metabolic and lipid index targets. A broad-based team of wellness professionals and academics evaluated workplace wellness programs. They unanimously concluded that few wellness programs meet expectations and most are abysmal failures. What separates bad, good, and great programs is “a combination of good design built on behavior change theory, effective implementation using evidence-based practices, and credible measurement and evaluation.” To further support the need for more thorough risk assessment, in a global study of 84 risks, the authors concluded “Increasingly detailed understanding of the trends in risk exposure and the relative risks for each risk-outcome pair provide insights into both the magnitude of health loss attributable to risks and how modification of risk exposure has contributed to health trends [9]. These types of data clearly illustrate a path to improved health outcomes through broader and deeper precision and personalized assessment.

The risk evaluation tool used in this study, the Chronic Disease Assessment™ (CDA), is an on-line health risk assessment and mitigation tool and involves answering approximately 120 questions that probe deeply into lifestyle and environmental sources of risks, behaviors, health attitudes, readiness to change, current and past complaints, problems and diagnoses. The output of the CDA is an overall risk score and then sub-sets of scores by risk categories, and a score for each question/answer combination. The overall raw risk score is converted into a letter “grade” reflecting the extent of the individual’s risk “portfolio.” The letter grade is provided to participants as an easily understood value for their risks, to overcome a lack of health literacy that especially impacts high risk populations. In addition, the CDA output generates a series of actions that provide personalized education and actionable solutions to each risk in a participant’s risk portfolio. Finally, a Health Revival Care Plan™ is generated from the risk portfolio, and adjusted by the health coach and the participant, to create a simplified, personalized roadmap to overcome risks and improve health.

A major impediment to health improvement is low health literacy. The de facto intervention perpetuating this problem is a prescription for symptom management that requires little knowledge by the patient. Deficits in health literacy are associated with poorer health outcomes and higher health-related costs for both individuals and systems. Improved health literacy has been associated with reductions in risk behaviors for chronic disease, and decreased rates of hospitalization [10]. Health literacy is a critical and under-examined component of health disparities. According to the National Assessment of Adult Literacy, over a third of U.S. adults have basic or below basic health literacy and have difficulty managing common health-related tasks. Limited health literacy poses a significant economic burden to our society, with national estimates indicating that low health literacy costs the U.S. healthcare system from $106 to $238 billion each year in 2007 healthcare dollars [11].

The nexus of this program, including the CDA risk portfolio, actions, care plans, and health coaching, is designed to meet and exceed the United States Department of Health and Human Services National Action Plan to Improve Health Literacy’s three goals: ensuring equitable access to health information; creating ‘person-centered health information and skills’ and supporting the development of the skills needed to attain and maintain good health. A final important aspect of this process, not articulated by the Action Plan, is illumination of the connection of risks to problems and complaints. The CDA collects and reports risks, problems, and complaints together. Thus, participants are able to “connect the dots” between risks and problems, like oral health and joint pain or carbohydrate intake and fatigue, as examples. These upstream-downstream connection realizations improve health literacy and stimulate more sustainable change which manifests in the adoption of actions and plans to eliminate the risks as a solution to their problems as opposed to the usual care option of a drug to control symptoms. This process empowers individuals to be a participant in their own health improvement through recognition of their control over causes and outcomes.

Within this study, health coaches interacted face-to-face and electronically with participants and groups of participants to implement care plans. Coaching activates patients to change through collaborative learning and social support. Patient engagement and 4P medicine, defined as predictive, preventive, personalized and participatory, is an increasingly important component of strategies to prevent and reverse chronic disease, at least within the functional and integrative medical communities. Interventions that tailor support to the individual’s level of activation, and that build skills and confidence, are effective in increasing patient activation. More highly activated people are more likely to engage in healthy behavior such as eating a healthy diet and getting regular exercise while avoiding health-damaging behavior such as smoking and illegal drug use. These behavioral changes have led to lower rates of hospitalizations and emergency department visits, compared to less activated patients [12].

A bridge between risk factors and modification in certain intermediate factors like blood pressure and obesity are changes to blood-based biomarkers, which are more objective measures of health. The most routinely performed tests in usual care are for the assessment of kidney and liver health, blood chemistry, lipid markers and metabolic markers. Heart disease continues to be the number one cause of morbidity and mortality in the U.S. and globally despite the broad use of cardiovascular disease medications for both prevention and intervention. A study of 136,905 patients hospitalized with a heart attack between 2000 and 2006 showed that almost 75% had LDL cholesterol levels within guidelines [13]. These data imply there is room for testing to augment evaluation of cardiovascular risk and cause. In older populations, “concentrations of homocysteine alone can accurately identify those at high risk of cardiovascular mortality, whereas classic risk factors included in the Framingham risk score do not” [14]. In healthy men, adding C-reactive protein levels to traditional risk factors, the Reynolds Risk Score, improved cardiovascular risk prediction. The Intermountain Risk Score uses common blood measures and assesses risk from the group of markers to develop a risk score. Although limited in application, this scoring system has been reported to be predictive of increased mortality risk and provides patients with a more definable goal, the improvement of the score.

More comprehensive assessments for risk and disease are emerging including the “Allostatic Load” and “Inflammaging” concepts. Each of these approaches considers a broader molecular view, rather than an organ system view of disease. According to McEwen, “When these (our body’s) adaptive systems are turned on and turned off again efficiently and not too frequently, the body is able to cope effectively with challenges that it might not otherwise survive. However, there are a number of circumstances in which allostatic systems may either be over-stimulated or not perform normally, and this condition has been termed “allostatic load” or the price of adaptation” [15]. Diabetes is a relevant example, where insulin production is frequently elevated in response to regular highly absorbable carbohydrate intake.

Claudio Franceschi coined the term “inflammaging” in 2000 to describe the concept of low-grade chronic inflammation and its impact on health. Inflammaging was described as an extension of the “network theory of aging” [16]. Similar to the allostatic load, a global reduction in the capacity to cope with a variety of stressors and a concomitant progressive increase in proinflammatory status are considered the major characteristics of the inflammation aging process and susceptibility to premature disease and mortality. Biomarkers for inflammaging are readily available and inexpensive but seldom obtain in usual care, especially in the implementation disease prevention strategies. According to Gay et al., the allostatic load leads to dysregulation of the neuroendocrine system and subsequent elevation in inflammatory markers, leading to metabolic syndrome and chronic diseases such as cardiovascular disease [17]. Thus, the allostatic load and inflammaging are both measured, at least in part, with inflammatory markers like C-reactive Protein, cortisol levels, glycosylated hemoglobin, white blood cell counts, and fibrinogen as examples.

Independent of inflammatory markers, multiple biomarkers, in general, improve the predictive power of a panel. In a study of 3209 people assessed with 10 biomarkers, persons with multi-marker scores in the highest quintile as compared with those with scores in the lowest two quintiles had elevated risks of death and major cardiovascular events of 4.08 and 1.84 (adjusted hazard ratios), respectively [18]. This far exceeds the predictive hazard ratio for cholesterol which varies from 0.89 to 1.25 depending upon the study [19]. A hazard ratio of < 1 means cholesterol levels were determined to be protective and stave off early mortality. Numerous studies and reviews consistently show the value of multiple markers in real-world prediction of disease events and premature mortality.

The Chronic Disease Temperature™ (CDT) risk scale used in this study combines emerging concepts for improving the evaluation of disease risk and measurement of active disease. The significant attributes of the CDT scale are:

1. consideration of multiple biomarkers,

2. selection of markers based on traditional and new predictive markers based on inflammaging and the allostatic load,

3. harmonizing each marker to a standard endpoint - increase in early mortality risk,

4. consideration of risk contribution based on log-linear deciles of marker levels and individual marker hazard ratios for mortality, and

5. combination of the risk values from each marker into a single number score to accommodate limited health literacy and to set an understandable objective target for health improvement.

The aggregate CDT score is an indicator of early mortality and associated total morbidity, while the values for each marker reflect both mortality risk and disease risk based on the association of a given marker to disease. This single number may be an important bridge to better health literacy as most patients do not understand the meaning of their current lab values. The CDT does not constitute a medical diagnosis of disease any more than does any individual marker, like homocysteine, but does statistically afford better predictive capability and measurement of disease progression or regression. The CDT output promotes the concept that health and disease lie on a log-linear continuum rather than being an on/off switch.

In this study, the implementation of risk assessment, health and disease measurement, care plans, and frequent measurement leading to continuous improvement represents a needed response to challenges society faces from chronic diseases. This “systems approach” is designed to better connect across fragmented divisions in healthcare without bias of discipline. That is, fundamentally, most chronic diseases are connected at root-cause physiological processes. The ultimate goal is to create new risk/plan/action/outcome connections that facilitate learning opportunities and iterative advancement in treatment and preventative methods for chronic disease. Another consideration is the order applied to the interventions including "in series" or "in parallel". An example is diabetes that needs to be managed for the prevention of heart disease, yet these diseases lie in different medical silos. The final objective ensures that workup of any individual patient, regardless of the presumed scope of the illness, embraces all possible causal factors.

The purpose of this study was to assess the effectiveness and safety of this novel care model for the prevention and reversal of a broad spectrum of chronic diseases and complaints over a nine-month period. Primary endpoints to assess the effectiveness of the intervention were changes in health risk assessment scoring, changes in documented health complaints, changes in medication usage, changes in vital signs, changes to individual blood-based biomarkers designed to measure chronic health, and changes to the multiple marker risk score, the CDT.

## Materials and methods

We conducted an open-label, randomized, controlled, before-and-after nine-month study of a high intensity remote and on-site care intervention named a health revival process (HRP). Participants included a group of 70 individuals who, at the time, were employed by a mid-west fortune 1,000 manufacturing company with approximately 1,000 employees at that site. No formal control group was established but non-participant health status over the period was tracked using claims data for diagnoses, complaints, medication use, and healthcare spending. Participation was voluntary and recruitment started in November of 2016, implemented by our company and the employer, focused on higher claims and more chronically sick individuals who were motivated to overcome unresolved chronic health issues. No rigid participation inclusion criteria were used other than each individual had at least one diagnosed chronic condition, was formerly or currently on a medication for a chronic disease, and was a high healthcare claimant (> $5000/year currently or within the past three years) if that data was available. Not all participants had claims data from previous years mainly due to their health plan and choice or employment history with the company.

From those interested in the program and met the criteria, retrospective health data (medical claims) were reconciled to finalize the 70-person cohort without consideration for a specific type of condition. Although not a formal clinical study, all procedures performed in the program involving human participants were in accordance with the ethical standards of the institutional and/or national research committee and with the 1964 Helsinki declaration and its later amendments or comparable ethical standards. Ethical oversight was provided by the existing primary care clinic management organization, but not under any formal written agreement other than to monitor for patient safety. Informed consent, medical releases, and participation contracts were obtained from all participants included in the program. These documents were completed after each participant was provided detailed information about the program. All data were acquired in strict conformance with health data privacy laws by medical personnel and all stored data were contained on a Health Insurance Portability And Accountability Act (HIPAA) compliant cloud.

The health revival process: Participants in the health revival process (HRP) underwent medical history review, completed a 120 +/- question online health risk assessment (Chronic Disease Assessment™ (CDA)) and laboratory testing for 55 serum-based biomarkers (Chronic Disease Temperature™ (CDT)). Participants without a medical exam within the past 6 months had one perform by our doctor, to determine their baseline health and risk status. All data obtained on participants, including problems, diagnoses, health complaints, reconciled medications, vitals, food journal, and other measurements were entered into our proprietary health revival software. Upon qualifying, HRP participants began one-on-one health coaching encounters. The initial coaching session included active listening by the coach and reconciliation between the output of the CDA risk assessment and health concerns, problems, and information articulated by the participant at that first encounter. Any discrepancy between the responses to the CDA questions and information presented to the health coach were verified and the CDA was appropriate updated and annotated. The health coach, using the recommendations promoted by the up-to-date HRP software record, developed a personalized health roadmap referred to as a person’s Health Revival Care Plan™ (HRCP). Our medical doctor reviewed and finalized that document and then conferred with the coach on next steps appropriate for the participant. Our doctor and the non-HRP medical doctors ensured that the recommendations and suggestions made by the health coach did not violate the health coaches license to provide advice. There was reasonable fidelity to the HRP delivery as everyone saw the same coach and doctor over the same time period. However, participants experienced different levels of coaching and doctor intensity based on the extent of their risk portfolio and medical needs.

**Figure 1:**
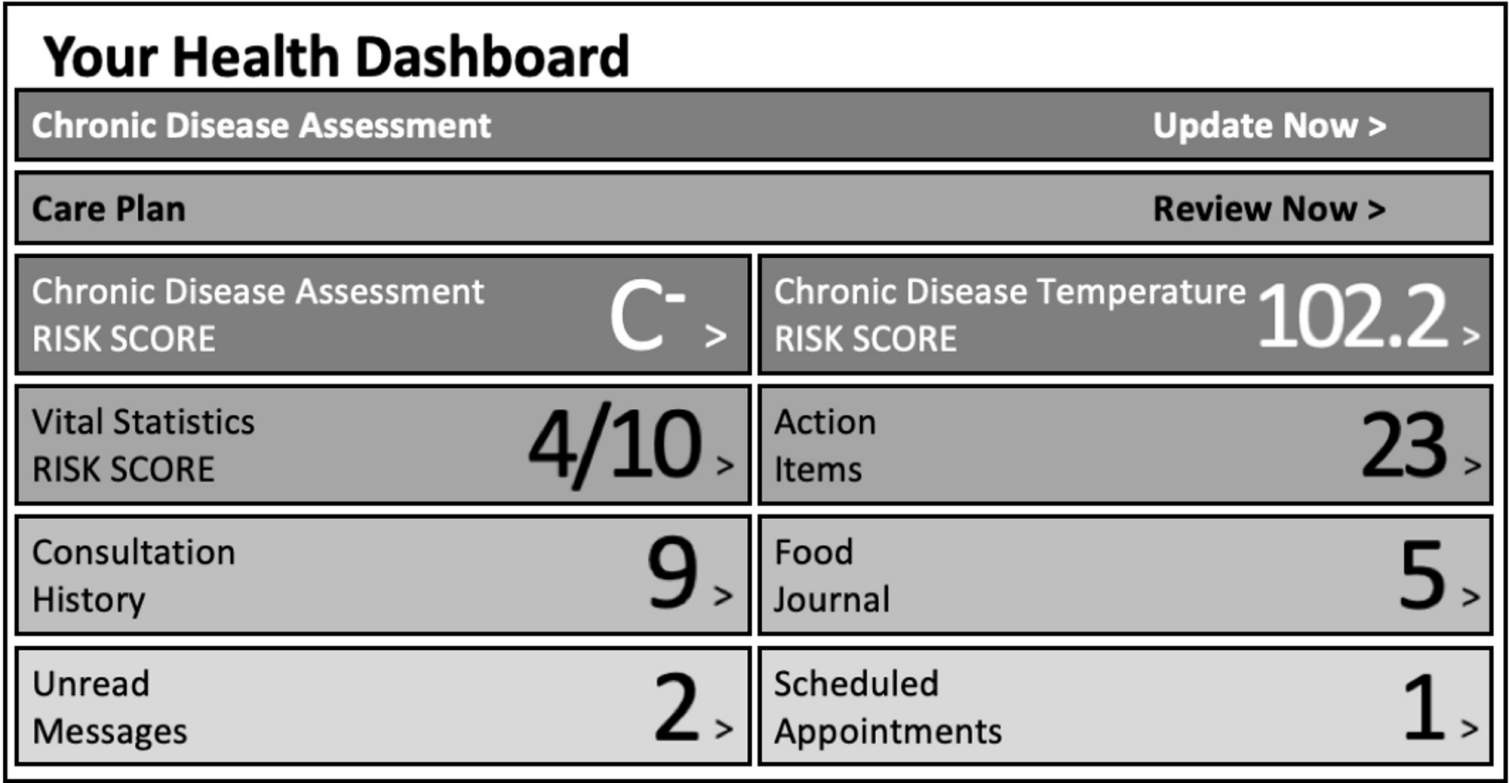
Patient Portal Home Page – Health Revival Dashboard

Participants had continuous access to their health information, suggestions, and progress within the HRP software which maintained much of their personal health information. Specifically, a participant could follow, track, and monitor changes in health measures and interact with specific, personalized content (written, audio, and video) curated automatically by the HRP system based on their personalized health information inputs including, their CDA risk assessment, CDT markers, and vital signs recorded in the system. In addition, the participant portal included their personalized HRCP that created the structure for their HRP. Participants were able to work with their health coach on the plan, follow the plan through a self-guided process, or some hybrid between the two paths. The health coach was able to monitor the self-guided process through system feedback that included reports on system logins and completed “actions” and “goals.” Completed actions occurred after a participant accessed content relevant to a health risk determined by the HRCP and choose a selection after reviewing the information like “completed” or “deferred.” Most participants relied on the health coach for structure, direction, and motivation. Figure 1 shows features of the patient portal Health Revival Dashboard.

Participants in the HRP retained their existing providers for acute disease management and routine medical checkups. Care coordination between existing providers and HRP care team occurred as needed. In particular, the HRP doctor and PCP discussed any possible interactions between supplements, lifestyle changes, and current medications. Of frequent concern was a need to change the dose or use of insulin, other diabetic medications, blood pressure, cholesterol, and corticosteroid medications as the participant’s health improved through lifestyle modification. Frequency and type of biomarker tracking, beyond the before-and-after CDT labs, were individualized by the HRP doctor on the basis of care needs and progress as recorded by participants in updated CDAs and health coaches in updated HRCPs and coaching notes. Participants on insulin were contacted at least weekly to assess any potential for hypoglycemia. Either the PCP or the HRP doctor made medication modifications.

**Figure 2:**
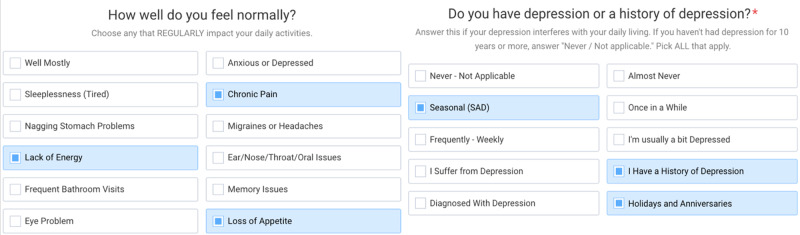
Example questions and answers from the Chronic Disease Assessment™ health risk assessment survey

Chronic Disease Assessment: The CDA was available electronically through a web browser and included 120+/- questions through a series of shorter surveys, with the participant able to stop and restart the survey at any point where they left off. Each question included fixed answer choices from a single option to as many as 30 options depending upon the scope of the question. In some instances, a question allowed for multiple answer selections while in others, only a single response could be recorded. Each question/answer combination was given a risk score and assigned one or more attributes of risk based on our study of the medical literature for potential outcomes associated with the specific behavior attribute. The CDA included consideration of common risks and disease root causes determined from our own clinical experience and published clinical case studies, population and randomized clinical trials. For example, the CDA gave considered to oral health, gut health, eye health status, macro- and micronutrient imbalances, and indications of chronic and occult infection. The output of the CDA was a raw score that converted to a letter “grade” reflecting the extent of the risk “portfolio” determined by the assessment. Each letter grade, A-F, spanned a range of raw scores. The purpose of the letter grade was to convert a numeric value into a more meaningful and recognizable risk level. In addition, the software interface outputted an action or a series of actions for many of the question/answer pairs, through if/then/else logic. The actions are those known or believed to lead to a reduction in that risk based on peer-reviewed published studies or our own experience. The actions are bundles of content that explain the risk or potential risks and offer suggestions to ameliorate the risk. All this content was made available to participants through their Health Revival Dashboard (HRD). Example questions from the CDA are provided in Figure 2.

Chronic Disease Temperature: Routine labs and the CDT biomarker panel were drawn at the on-site clinic by non-HRP technicians working for the primary care provider company. The labs drawn on each individual and the thresholds for chronic health considerations are described elsewhere [20]. In summary, reference intervals were not used to determine health status and risk. Instead, our team of biostatisticians reviewed the medical literature to determine the threshold level or levels for each biomarker, they indicated a statistically significant increase in early mortality associated with that marker. Each marker was assigned escalating risk based on log-linear curve fitting to published information on mortality risk and biomarker raw value. Many of the CDT biomarkers are common biomarkers with some being less commonly obtained in usual care. The ordering doctor of record was held responsible for ensuring any participant with an abnormal lab value, based on usual care reference intervals, notified the participant and arranged for appropriate care to correct the abnormal value. The “tighter” thresholds used as part of the CDT labs were used only by the HRP doctor and not used for making a medical diagnosis. Instead, this more sensitive scale of normal vs abnormal lab values was used to measure changes in participant’s physiology concomitant with lifestyle changes. This more sensitive scale for each biomarker and the overall CDT value, helped assess participant’s health trends by recognizing that disease is not an on/off switch, but rather a continuum. For example, and HbA1C level of 6.3%, although not a diagnosis for type 2 diabetes, is a strong indicator of future type 2 diabetes. A goal value of < 5.0% was established for all participants to optimize insulin sensitivity and avoid future diabetes risk, assuming that value was obtained through lifestyle improvement and not pharmaceutical intervention. This logic was applied to all CDT markers.

The interventions affected by the HRP through the HRCP were individualized to each participant and included a consideration of the participant’s readiness to change and the likely sustainability of any given change as determined by their responses to Chronic Disease Assessment questions and discussions with their health coach. The intensity of coaching was predetermined, but not fixed, by the CDA grade. The coaching time allotted to each participant based on the CDA grade is provided in Table 1.

**Table 1:** Heath coaching time allocated to a participant based on their Chronic Disease Assessment letter grade

CDA Letter Grade	Health Coaching Time Allotted Annually (hours)
A	2
B	6
C	10
D	16
F	20

Doctor time allocation was approximately 1/10th coaching time. The main risk considerations were a reduction in inflammation through: more movement, increased nutrient density of foods, improvement in digestion/absorption by improving gut balance and activity, increasing probiotic and prebiotics foods, elimination of high glycemic foods, better oral health maintenance, increased intake of healthy fats and omega-3 fatty acids, increased micronutrients to support hormone production, stress reduction, brain health through reducing whole-body inflammation, and consumption of greater amounts of fat-soluble vitamins, as examples. No specific nutrition program was recommended to the participants as a whole. Instead a simple process of substitution of one food, considered of low nutrient value for another of higher value, was made as recommended by the health coach. Recommendations were made on an individualized basis based on participant preferences, to affect a gradual and sustainable change from the Standard American Diet (SAD) that was prolific throughout the cohort, to a new food consumption pattern with increased micronutrient density, fiber, and fat with less carbohydrate and sugar consumption. Short term (one to three months) nutritional ketosis was suggested for a few highly insulin resistant and diabetic patients but no participants fully achieved nutritional ketosis during the nine-month period. However, these participants achieved a significant reduction in total carbohydrate consumption with a shift to low glycemic index food and those containing higher concentrations of marine, monounsaturated, and saturated fats.

Supplements were provided as part of the program and compliance with supplementation was near 100% based on self-reporting and resupply orders. At the outset of the program, after evaluation of CDT labs, CDA reported dietary information, and food journals, the participants were provided any or all of the following supplements based on individually assessed deficiencies: multivitamin/mineral (1 QD); Vitamin D3 (2000-10,000 IU, QD); cod liver oil (5-15 g, QD); Magnesium Glycinate ( 100-400 mg, QD); Vitamin K2 (50 mcg, QD); probiotic (20-70 billion organisms, QD). Supplements were provided only when deemed appropriate by the HRP doctor and were phased in then phased out over the nine-month period as the doctor determine that nutritional deficiencies or insufficiencies were being mitigated by the program. The purpose of the supplements was to quickly overcome apparent nutritional deficiencies. As part of the HRCP, foods that contained nutrients provided by the supplements were recommended and, when adopted, enabled the gradual elimination of supplementation without compromising nutritional needs. The main behavioral change strategy, executed by the doctor and coach care team, was to slowly and gradually ease a participant into change. The frequency of coaching sessions, dictated by the need of the participant (CDA letter grade), including their burden or disease and risk, and their motivation, was adjusted to improve compliance. Most participants suffered from some health ailment that impacted their daily wellbeing. Improvement in general wellbeing, which started to be noticed by participants by the end of month 1, provided the motivation to continue to adopt gradual modifications to lifestyle. To help participants understand that the HRP is not a quick fix, our coaches explained that we have determined a general “rule of thumb” for the time required to improve health significantly. If a disease, like diabetes, has been slowly developing over five years, it will take at least five months of effort to reverse the disease, assuming the interventions are appropriate.

Outcome Measures: In-clinic vital signs, health risk assessment (CDA) risk score and list, and biomarkers were obtained at baseline and at the end of the 9-month program. Problems, complaints and medications were reconciled at each health coaching and doctor encounter. Fasted and non-fasted blood draws were obtained by clinic PCP staff using routine procedures. Samples were provided to and analyzed by Quest Diagnostics using standard operating procedures. Primary outcomes were: changes in biomarker values; risk scores; reported diagnoses; vital signs; weight; and medication use. Secondary outcomes included reported complaints, for example, lack of energy, chronic pain, sleeplessness, mood issues, and general lack of wellbeing.

## Results

The baseline demographics of the final 70 HRP participants are presented in Table 2. All participants were Caucasian of European heritage. At baseline, 96% of HRP participants were actively taking pharmaceuticals for a medical problem and 98% were diagnosed with at least 1 chronic condition. This reflects a substantially higher percentage of chronically ill individuals compared to the U.S. national average of 60% of U.S. adults having at least one chronic condition. On average, the group was taking 2.3 prescriptions per person. The major class of medications included: diabetes medications, injectable insulin, statins, blood pressure lowering, pain, mood (SSRIs), bisphosphonates, steroids, thyroid hormone, and proton pump inhibitors. The final participant number of 70 was established after 10 left the program. Two were dismissed from the program for compliance reasons, four left early to join a weight loss program, two left because of the time commitment, and two left due to potential interactions between current medications and supplements as encouraged by their PCP, for a total of 12.5%. There was no clear demographic trend between those who remained in the program versus those who dropped out.

**Table 2:** Demographics and chronic condition status for 70-participant cohort

Demographics	Male	Female	Total
# Participants (%)	20 (29%)	50 (71%)	70
Average Age	53	52	53
20-29 (% of gender)	0 (0%)	2 (4%)	2 (3%)
30-39 (% of gender)	3 (15%)	4 (8%)	7 (10%)
40-49 (% of gender)	4 (20%)	14 (28%)	18 (26%)
50-59 (% of gender)	7 (35%)	17 (34%)	24 (34%)
60 -69 (% of gender)	6 (30%)	12 (24%)	18 (26%)
70-79 (% of gender)	0 (0%)	1 (2%)	1 (1%)
College Degree (% of gender)	23 (46%)	5 (25%)	28 (40%)
Some College (% of gender)	7 (14%)	1 (5%)	8 (11%)
High School or Below (% of gender)	19 (38%)	14 (70%)	33 (47%)
Diagnosed Chronic Conditions	Male	Female	Total
0 (% of gender)	2 (7%)	3 (8%)	5 (7%)
1 (% of gender)	6 (20%)	10 (25%)	16 (23%)
2 (% of gender)	7 (23%)	9 (23%)	16 (23%)
3 (% of gender)	8 (27%)	11 (28%)	19 (27%)
4 (% of gender)	5 (17%)	5 (12%)	10 (14%)
5+ (% of gender)	2 (7%)	2 (5%)	4 (6%)

**Figure 3:**
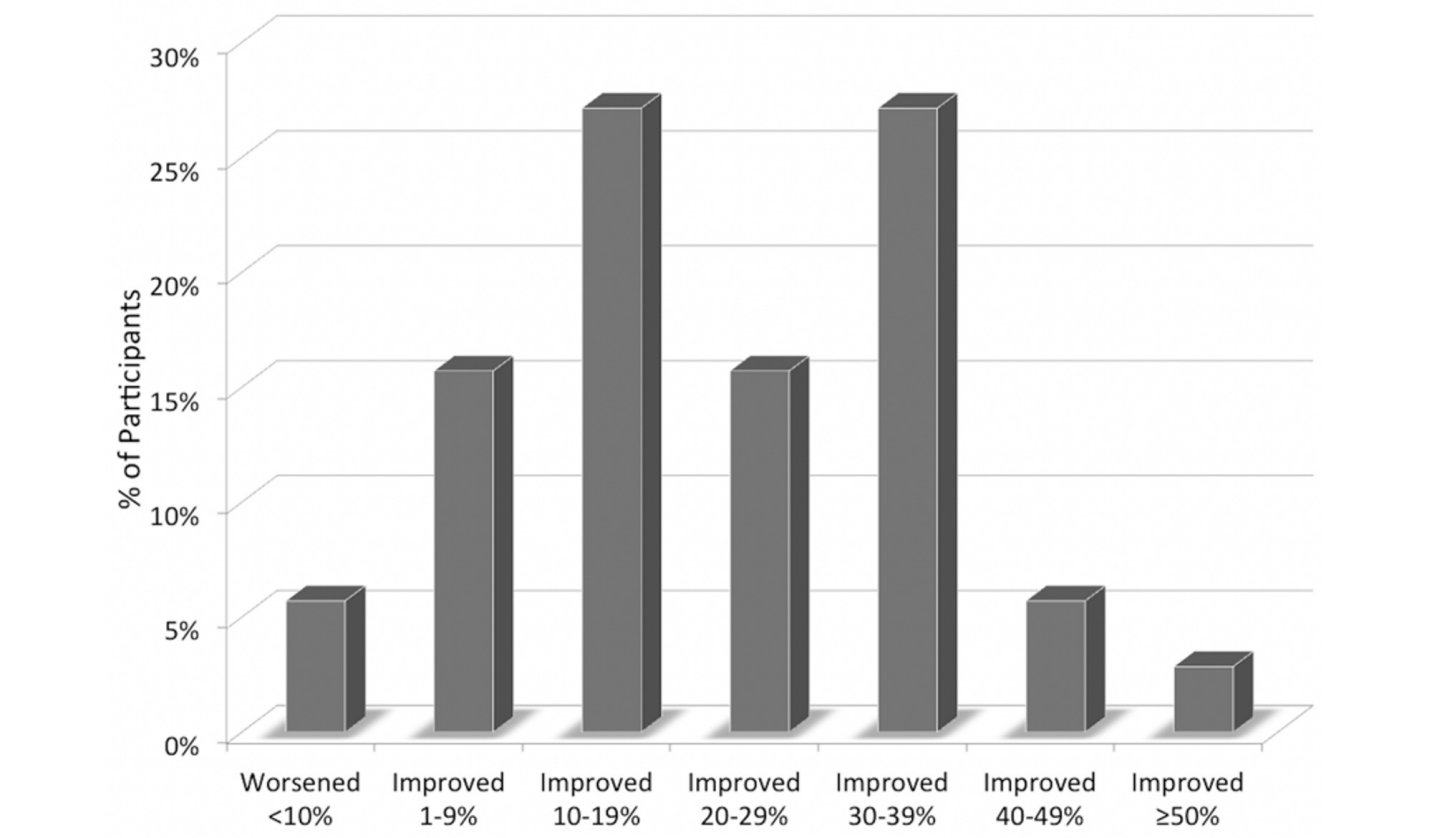
Change in the Chronic Disease Assessment risk grade by % range

Chronic Disease Assessment™ (CDA): On average, the 70-participant cohort lowered their CDA score by 31 points (27%) from a raw value of 115 to a new value of 84 over six months. Each point lowered reflected a reduction in a disease risk or resolution of a health problem or complaint. Risks were scored on a 1-10 scale, with 1 representing a minor risk or problem and 10 representing a major risk or problem. The initial population CDA grade was D+, assigned based on a range of raw numeric scores calculated from the survey, and the final grade after 6 months of the HRP was C+. Ninety-four percent of the group experienced an improvement in their risks while 6% of the cohort experienced a worsening of their CDA grade (Figure 3). Participants did not have their initial CDA answers to refer to when they retook the assessment six months into the program.

**Figure 4:**
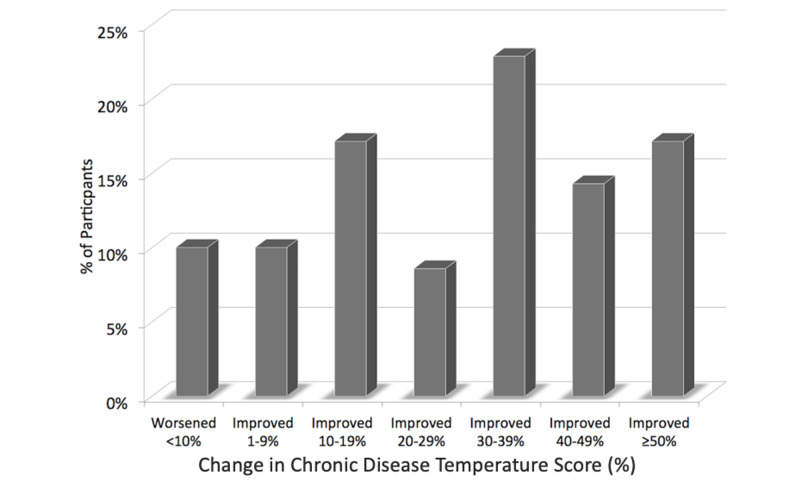
Change in Chronic Disease Assessment score (%)

Chronic Disease Temperature™ (CDT): On average, the 70-participant cohort lowered their CDT score from 102.1 to 100.8. The CDT is based on a 7 “degree” scale calculated by adding the risk contribution from each biomarker to 98.6 to arrive at the participant’s CDT. The average chronic disease risk reduction in CDT was 39% (Figure 4).

All 6% of participants whose CDA grade worsened also experienced an adverse change in their CDT score. Three participants improved their reported CDA grade but witnessed an adverse change in their CDT. The CDT included several markers that are classified as “acute phase” reactants. In "Acute-phase proteins and other systemic responses to inflammation,” the authors explain that markers of chronic systemic inflammation are also subject to change acutely [21]. For example, C-reactive protein elevates during the acute phase of pneumococcal pneumonia. C-reactive protein has an acute phase relaxation half-life of approximately one day upon removal of the insult whereas the half-life of fibrinogen is about one week. Two of the three participants with an improved CDA but worsened CDT experienced adverse physiological changes due to documented acute circumstances. Retest was not available during the nine-month program to confirm our suspicion about the cause of the elevation. One recently underwent surgery and was recovering slowly. Another participant was receiving ongoing treatment for a complex acute condition managed by the patient’s PCP.

Other participants whose CDT worsened were in the HRP and a calorie-restricting weight loss program administered by third parties. The weight loss program was calorie counting-based with no guidance provided on macro and micronutrients. These individuals, although in the HCP, were less flexible to our coach’s dietary suggestions because of the calorie restriction. For example, a participant refused to take cod liver oil because each capsule contributed approximately 10 calories to their daily calorie allotment. Our results demonstrated, in this small subgroup, that people in poor health and with a highly elevated CDT, confirming their health status objectively, may be contraindicated for a sustained calorie restriction program without nutritional guidance. Although the literature is rich in studies suggesting that calorie restriction improves lifespan and reduces inflammatory markers, emerging studies emphasize that calorie restriction must be implemented without malnutrition that comes from low nutrient-dense foods processed foods. Macro- and micronutrient intake of all participants was monitored with a food journal. Worsening in CDT markers in people on a calorie-restricted diet correlated to micronutrient malnutrition exacerbated by reduced total calorie intake. Malnutrition status was established in these individuals by determining their daily nutrient recommendation from the DRI calculator for healthcare professionals provided by the USDA and comparing the results to nutrients consumed based on available nutritional labels for foods consumed.

Relationship between changes to the risk measurements (CDA) and blood markers (CDT)

**Figure 5:**
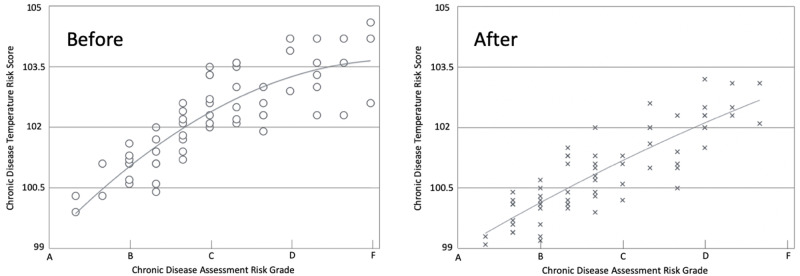
Correspondence between lifestyle risks (Chronic Disease Assessment grade) and blood-based biomarkers (Chronic Disease Temperature score) before and after six months of the HRP program

**Figure 6:**
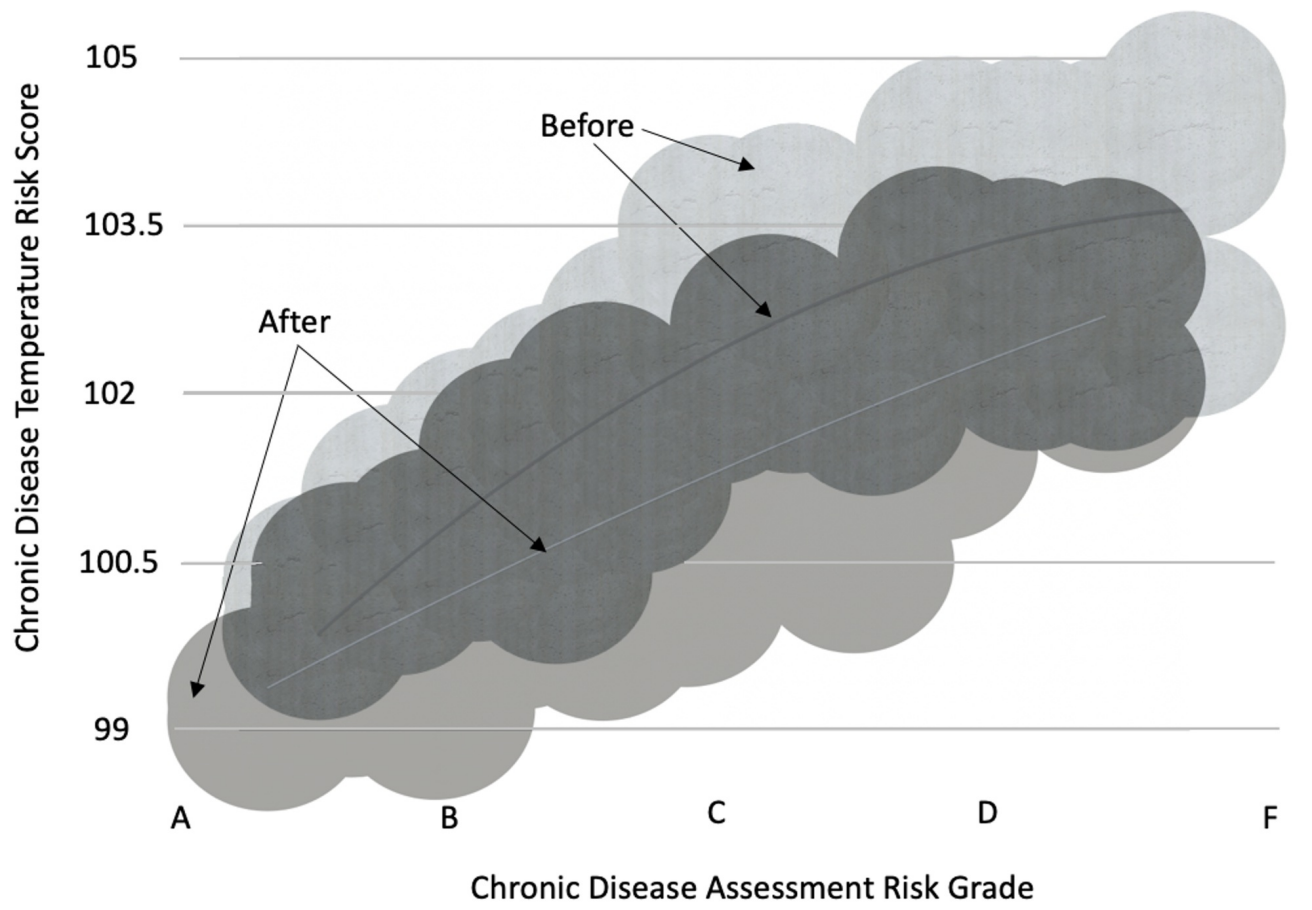
Correspondence between lifestyle risks (Chronic Disease Assessment grade) and blood-based biomarkers (Chronic Disease Temperature Score) before and after six months of the HRP program - overlay chart

There are numerous studies on the association between lifestyle behaviors and chronic disease risk. In large prospective studies, like the Nurses’ Health Study, vague conclusions are made about the association of smoking, regular physical activity, maintaining normal body mass index, eating a healthy diet and chronic disease proliferation [22]. The individualized CDA risk values potentially increase the precision, personalization, and accuracy of risk-to-disease relationship measurement. Figure 5 provides a view of the change in the CDA risk score and its relationship to the CDT value for the biomarker panel at the beginning and end of the 9-month HRP program for the entire population. The same data are presented in Figure 6 as bubble chart with the before and after data superimposed on the same scale.

Notable is the reasonably smooth relationship between the two risks scores, the subjective CDA and the more objective CDT. We conclude, from these data, reducing the most basic health risks, over time, may lead to a reduction in cytokine burden with often concomitant change in diagnosed chronic diseases. Increasing the “n” in our database and making appropriate adjustments to assigned subjective risk values within the algorithm offers the potential to improve the correlation between determinants of health risks and physiological health status. The individualized statistics for the CDA, CDT, and biomarkers comprising the CDT to evaluate participants health and risk are provided in Table 3.

**Table 3:** Chronic Disease Assessment risk score and grade, Chronic Disease Temperature score, and individual biomarker values before and after the health revival program (HRP) CDT is the Chronic Disease Temperature biomarker score as a relative value with 98.6 considered optimal and elevated values indicating chronic risk; HbA1C is expressed as a %; Glucose is expressed as mg/dL; NLR is the neutrophil to lymphocyte ratio; hs-CRP is high sensitivity C-reactive protein or C-reactive protein, cardiac expressed as mg/L; insulin is expressed as uIU/mL; HDL is expressed as mg/dL; triglycerides are expressed as mg/dL; vitamin D is expressed as ng/mL; uric acid is expressed as mg/dL; WBC is the white blood cell count expressed as cells/uL; RDW is the red blood cell distribution width expresses as a %; Ab neutrophils are neutrophils (absolute) expressed as cells/uL; ESR is the sedimentation rate-Westergren expressed in mm/hr; fibrinogen is fibrinogen activity expressed as mg/dL; homocysteine is expressed as umol/L; and AIP is the Atherogenic Index of Plasma expressed as the log(Triglycerides/HDL)

Biomarker / Risk Score	Mean Before	Mean After	Mean Difference	Standard Deviation	T Test Value	P-Value
CDT	102.1	100.8	1.28	0.79	7.89	<0.0001
HbA1C	5.87	5.17	0.72	0.45	9.25	<0.0001
Glucose	104.1	95.1	9.01	16.08	1.69	0.0480
NLR	2.2	1.9	0.29	0.92	2.45	0.0083
hs-CRP	2.21	1.77	0.44	1.98	1.86	0.0338
Insulin	10.6	7.1	3.52	5.82	3.65	0.0003
HDL	57.5	61.7	4.23	13.25	2.67	0.0047
Triglycerides	124	101	23	71	1.59	0.0578
Vitamin D	38	60	22	17	10.8	<0.0001
Uric Acid	5.18	5.19	-0.1	0.99	-.0130	------
WBC	6430	5900	530	1525	2.33	0.0113
RDW	13.8	13.0	0.82	1.00	6.84	<0.0001
Ab Neutrophils	3930	3380	552	1270	2.99	0.0020
ESR	10.5	7.1	4.43	7.58	1.58	0.0598
Fibrinogen	301	285	16.4	61.05	0.77	0.2221
Homocysteine	8.91	9.32	-0.40	2.39	-1.42	------
AIP	0.26	0.20	0.07	0.31	1.84	0.0349
CDA Risk Score	115 (D^+^)	84 (C^+^)	31.2	26.30	8.21	<0.0001

Population case studies

Glycosylated hemoglobin (A1C): A1C, a 120-day retrospective average of blood glucose, contributes to an assessment of metabolic risk along with fasting glucose and insulin. A current therapeutic goal in usual care is to lower the A1C value of diabetics, those with A1C values above 6.4%, with pharmaceuticals. The ACCORD study shows that tight pharmaceutical control of blood sugar in those with severe insulin resistance suffer a significant increase in adverse cardiovascular events and mortality compared to those with less tight control [23]. Lifestyle interventions offer another approach to glycemic control and does so without risk of hypoglycemia and other side effects of the pharmaceutical approach. In the cohort of 70, none had optimal A1C levels, defined as 4.2%-5.2%. Even a 0.5% increase in A1C above 5% increases the 6-year risk for diabetes (odds ratio >1.5) and the risk of diabetes increases exponentially with A1C. In the 70-participant cohort, at the end of the HRP, 98% (all but 1) lowered their A1C value (Table 4).

**Table 4:** Before and after hemoglobin A1C levels for the 70 participants and associated changes in metabolic state

A1C Status	Interpretation	A1C Initial	A1C Final
> 6.4%	Type 2 Diabetic	9 (13%)	0
5.7% – 6.4%	Pre-Diabetic	29 (41%)	8 (12%)
5.1% – 5.6%	Insulin Resistant	32 (46%)	37 (54%)
≤ 5%	Insulin Sensitive	0	24 (34%)

Insulin is the most sensitive marker for early metabolic risk because it increases first as an individual becomes insulin resistant. Even values slightly above normal, and well below a diagnosis of diabetes, contribute to serious chronic diseases in the future, including Alzheimer’s and cardiovascular disease. Type 2 diabetes is associated with increased risk of cancer. Hyperinsulinemia (elevated insulin levels) and insulin resistance are apparently the link. In a 15-year mortality study, individuals in the highest quintile of serum insulin had a 62% higher risk of cancer mortality and a 161% higher risk of gastrointestinal cancer mortality [24]. The authors of this study concluded that hyperinsulinemia/insulin resistance is associated with cancer mortality independently of diabetes, obesity/visceral obesity and metabolic syndrome.

In the 70-person cohort, 38 participants (54%) were at elevated metabolic and associated chronic risk. Six of the 38 (16%) experienced a double digit drop in fasting insulin, 8 of 9 (89%) dropped from the high-risk category to a lower risk level, 17 (52%) lowered insulin levels sufficiently to reduce their cancer risk severity category, 11 (33%) changed little and stayed in the same risk category and 2 move up one risk category (Table 5).

**Table 5:** Before and after insulin levels for the 70 participants and associated changes to metabolic and cancer risk

Fasting Insulin Status	Interpretation	Insulin Initial	Insulin Final
<6 uIU/ml	Very Low Risk	32 (46%)	54 (77%)
6 – <10 uIU/ml	Low Risk	15 (21%)	7 (10%)
10 – <20uIU/ml	Moderate Risk	14 (20%)	8 (12%)
≥20 uIU/ml	High Risk	9 (13%)	1 (1%)

C-reactive protein (CRP) is an important and independent biomarker for vascular disease risk and premature mortality. CRP, an acute phase protein, is synthesized by hepatocytes in response to inflammatory cytokines. Ridker et al. indicate that CRP is much more predictive of adverse cardiovascular outcomes compared to cholesterol since more that > 50% of people who experience a first heart attack have normal to low total cholesterol levels [25]. In the 70-person cohort, 56% of participants who were at high risk for cardiovascular disease, based on hs-CRP level >5, lowered that risk (Table 6).

**Table 6:** Before and after hs-CRP levels for the 70 participants and associated changes to cardiovascular risk hs-CRP is high sensitivity C-reactive protein or C-reactive protein, cardiac

hs-CRP Status	Interpretation	hs-CRP Initial	hs-CRP Final
< 0.5 mg/L	Very Low Risk	20 (29%)	26 (37%)
0.5 – 2 mg/L	Low Risk	25 (36%)	24 (34%)
> 2 - 5 mg/L	Moderate Risk	16 (23%)	16 (23%)
> 5 mg/L	High Risk	9 (13%)	4 (6%)

Atherogenic Index of Plasma (AIP) is a vascular risk parameter based on the log of the ratio of triglycerides to HDL. People with AIP values above 0.24 have increased premature mortality compared to people with lower AIP values. Edwards et. al. showed a 29% increase in all-cause mortality for people with an AIP of > 0.24 compared with those at level ≤ 0.24 [19]. The AIP average value before the HRP was 0.26 and lowered to 0.20 at the end of the HRP.

Conventionally the RDW test, which is a part of a complete blood count, is used to help determine anemia status. However, it is also a marker of inflammation and often tracks with CRP. Red blood cells elongate and deform when flowing through capillaries, which may explain the association between red blood cell widths, vascular inflammation, and increased cardiovascular morbidity and mortality. In the 70-person cohort, 92% of participants at elevated risk for cardiovascular disease based on RDW levels lowered their risk (Table 7).

**Table 7:** Before and after red blood cell distribution widths (RDW) for the participants and associated changes to cardiovascular risk

RDW Status	Interpretation	RDW Initial	RDW Final
< 12.5%	Very Low Risk	2 (3%)	21 (30%)
12.5 - < 13.5%	Low Risk	23 (33%)	35 (50%)
13.5 – 15%	Moderate Risk	36 (51%)	12 (17%)
> 15 %	High Risk	9 (13%)	1 (1%)

White blood cell counts (WBC) is a predictor of strokes, heart attacks, and fatal heart disease. In the Women’s Health Initiative involving 72,242 women from 50-79 years of age, those with approximately 6,700 white cells per mL had more than double the risk of fatal heart disease than women with 4700 cells per mL [26]. White blood cell counts in the normal range for acute indications are now more widely recognized as a predictor of adverse chronic outcomes.

In the 70-person cohort, three individuals had high cardiovascular disease risk based on WBC levels, and 100% lowered that risk through lifestyle modifications. In addition, 54% of those with moderate risk moved to either low or very low risk as assessed by risk quartiles for WBC. In general, 80% of participants moved from a high to a lower risk status (Table 8).

**Table 8:** Before and after WBC for 70 HRP participants and associated change to cardiovascular risk

White Blood Cell Status	Interpretation	White Blood Cell Initial	White Blood Cell Final
< 4000 cells/µL	Escalating Risk	0	0
4000-6000 cells/µL	Very Low Risk	26 (37%)	46 (66%)
> 6000-7800 cells/µL	Low Risk	27 (39%)	18 (26%)
> 7800-10,000 cells/µL	Moderate Risk	13 (19%)	6 (9%)
> 10,000 cells/µL	High Risk	3 (4%)	0

Multiple studies show a significant inverse relationship between 25-hydroxy vitamin D (D3) status and cancer mortality. In a fifteen-year study of nearly 50,000 participants, an increment increase of 10 ng/ml was associated with a 17% reduction in total cancer incidence, 29% reduction in total cancer mortality and statistically significant reductions in colorectal, pancreatic, esophageal, oral, and pharyngeal cancer mortality [27].

For cancer, optimal D3 levels are above 55 ng/ml. At the start of the program, eight participants had optimal levels and that number increased to 35 by the end of the program. Insufficient vitamin D, as defined for bone health are values below 30 ng/ml. Initially there were 14 participants insufficient for blood D3 and none were insufficient at the end of the program. The population D3 levels went from 38 to 60 ng/ml, on average, by the end of the HRP. These data indicate a high degree of compliance with the program recommendations as the increase in D3 status was largely attributable to consistent supplementation. In general, the increase observed required daily supplementation of 5000 IU D3 daily (Table 9).

**Table 9:** Before and after 25-hydroxy vitamin D (D3) levels for the participants and associated changes to cancer risk

Vitamin D Status	Interpretation	Vitamin D Initial	Vitamin D Final
> 55-100 ng/ml	Very Low Risk	7 (10%)	35 (50%)
45-55 ng/ml	Low Risk	9 (13%)	25 (36%)
30-45 ng/ml	Moderate Risk	39 (56%)	10 (14%)
< 30 ng/ml	High Risk	15 (21%)	0

The neutrophil-to-lymphocyte ratio (NLR) is reported to be a robust outcome prognosticator in existing solid tumor cancers. In a study on breast cancer, patients with an NLR > 3.3 had substantially higher one-year and five-year mortality rates compared to those with an NLR < 1.8. The NLR value has similar predictive ability for cardiovascular mortality [28]. In the cohort of 70, 20 had NLR above the threshold for adverse cancer outcomes. Sixteen of 20 (80%) saw their NLR ratios return to very low risk (normal values) by the end of the program (Table 10).

**Table 10:** Before and after neutrophil-to-lymphocyte ratio (NLR) levels for 70 HRP participants and associated changes to cancer and cardiovascular mortality prognosis

NLR	Interpretation	NLR Initial	NLR Final
< 2.5	Very Low Risk	50 (71%)	65 (93%)
2.5 - < 3.3	Low Risk	14 (20%)	4 (6%)
3.3 - < 5	Moderate Risk	4 (6%)	1 (1%)
≥ 5	High Risk	2 (3%)	0

Medication prescription reduction was achieved as part of the outcome measurement. The cohort experienced a 32% reduction in medication usage, reduction in dose in 8%, and an avoidance of two costly medications. The prices included in Table 11, below, where the actual pharmacy costs realized by the health plan and did not include any co-pay.

**Table 11:** Reduction in and avoidance of medication usage as a result of the nine-month health revival program (HRP)

Medications Eliminated	Purpose	Count	Cost/YR 1 Dose	Cost/YR
Atenolol	Blood Pressure	2	$243	$486
Spironolactone	Blood Pressure	1	$350	$350
Lisinopril	Blood Pressure	3	$292	$876
Hydrochlorothiazide	Blood Pressure	2	$574	$1,148
Omeprazole	Acid Reflux	6	$925	$5,550
Lansoprazole	Acid Reflux	3	$782	$2,346
Prozac	Antidepressant	1	$11,337	$11,337
Lexapro	Antidepressant	2	$1,597	$3,194
Zoloft	Antidepressant	1	$4,812	$4,812
Zyrtec	Allergy	1	$547	$547
Ranitidine	Allergy	2	$727	$1,454
Loratadine	Allergy	2	$346	$692
Flonase	Allergy	1	$365	$365
Albuterol	Asthma	1	$148	$148
Furosemide	Fluid Retention	1	$47	$47
Topamax	Migraines	1	$3,991	$3,991
Ibuprofen	Pain	3	$58	$174
Azathioprine	Autoimmune	1	$10,223	$10,223
Colace	Constipation	2	$256	$512
Calcium	Bone Density	3	$85	$255
Ferrous Sulfate	Iron	1	$29	$29
Alendronate	Bone Density	2	$1,851	$3,702
Tamsulosin	Urinary Retention	1	$97	$97
Insulin	Diabetes	1	$736	$736
Ambien	Sleep	1	$6,886	$6,886
Atorvastatin	Cholesterol	1	$154	$154
Simvastatin	Cholesterol	3	$219	$657
Crestor	Cholesterol	2	$6,692	$13,384
Subtotal				$74,152
Medications Avoided	Purpose	Count	Cost/YR 1 Dose	Cost/YR
Enterecept	Psoriasis	1	$67,573	$67,573
Adalimumab	Rheumatoid Arthritis	1	$61,128	$61,128
Subtotal				$128,701
Medications Reduced	Purpose	Count	Cost/YR 1 Dose	Cost/YR
Naproxen	Pain	3	$401	$602
Lisinopril	Blood Pressure	3	$292	$438
Paxil	Anti-Anxiety	1	$2,380	$1,190
Simvastatin	Cholesterol	5	$219	$548
Imitrex	Migraines	1	$22,792	$11,396
Prednisone	Inflammation	1	$166	$139
Subtotal				$14,173
Total Savings				$217,026

The cohort experienced a reduction in chronic disease burden. Chronic disease reduction was determined by changes in any of the following: actual change to a medical diagnosis, elimination of a medication associated with an existing diagnosis, changes in a vital sign that indicated a migration out of a diagnosis that was affected without the use of a medication, or change in a biomarker value or values that were initially used to make the diagnosis into a “normal” range without the use of medications (Table 12).

**Table 12:** Reduction in chronic disease burden from the nine-month health revival program (HRP)

Diagnosed Chronic Conditions	Total Before	Total After
0 (%)	1 (2%)	31 (44%)
1 (%)	15 (21%)	18 (26%)
2 (%)	19 (27%)	14 (20%)
3 (%)	20 (29%)	6 (9%)
4 (%)	11 (15%)	1 (1%)
5+(%)	4 (6%)	0 (0%)

Individual case studies

Case 1: Debilitating Psoriasis - 62-year-old female seamstress with high school education (Table 13).

**Table 13:** Case 1: Participant Chronic Disease Assessment (CDA) Grade and Chronic Disease Temperature (CDT) score before and after nine months in the health revival program (HRP)

Marker	Before	After
CDA	D	C
CDT	101.9	99.9

This participant presented with major risk factors and complaints including lack or exercise, fast food diet, high carbohydrate diet, daily high fructose corn syrup containing beverage consumption, use of omega-6 containing oils in cooking, statin drug daily prescription for primary cardiac event prevention, severe arthritis, psoriasis, and cataract. The severity of the psoriasis and her job function put her at risk of imminently going on disability. She had seen multiple specialists, was placed on antihistamines and topical steroids but her psoriasis condition continued to worsen. The next treatment option for her was to be etanercept which she declined pending the outcome from the HRP. She indicated that she had not washed her hands without pain in over a year. The HRP included 45-minute semi-monthly health revival coaching following the participant and care team agreed upon care plan. Cholesterol-lowering drug usage was eliminated in the first month as directed by our medical director. Health coaching focused mainly on food substitutions, increasing activity, value and use of supplements, a limited set of supplements, and additional care to her oral hygiene.

**Figure 7:**
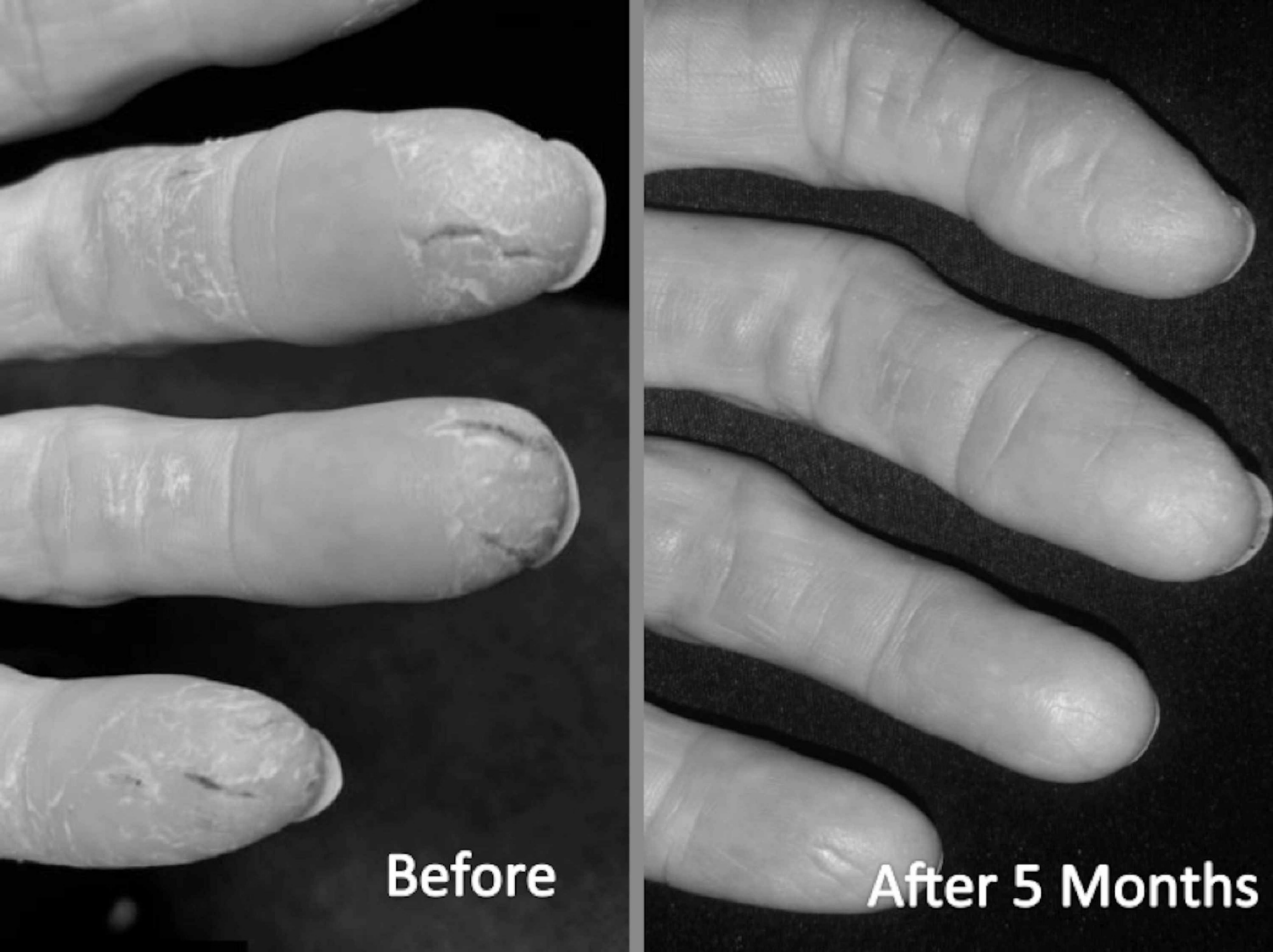
Visual change in severe psoriasis after five months in health revival program (HRP) Photographs obtained by the participant without prompting

After six months of intensive health revival coaching, many risk factors and complaints, revealed on her CDA report, were either removed or reduced including nagging chronic pain. Her main complaint, debilitating psoriasis slowly, but completed resolved in five months (Figure 7). However, the first signs of improvement in her psoriasis condition did not appear until month 4 of the program.

Normally, in the case of autoimmune diseases like psoriasis, food sensitivities or allergies must be addressed. This participant was unwilling to eliminate some of the common allergens like gluten and dairy. She was placed on a modest supplement regiment based on nutritional deficiencies determined from food journaling, including: cod liver oil (5 g/day); vitamin D3 (5,000 IU/day) and a multivitamin/mineral supplement (taken per label instruction), and the other general supplements included in the “Methods” section. Positive changes in lab values included: 25-hydroxy vitamin D status (24 to 55 ng/ml); white blood cell counts (6,200 to 5,500); RDW (14.6% to 13.0%); and fibrinogen (339 to 282 mg/dL).

Case 2: Rheumatoid arthritis and type 2 diabetes - 62-year-old male factory worker with a high school education (Table 14).

**Table 14:** Case 2: Participant Chronic Disease Assessment (CDA) Grade and Chronic Disease Temperature (CDT) score before and after nine months in the health revival program (HRP)

Marker	Before	After
CDA	D^+^	B^-^
CDT	102.5	100.8

This participant presented with major complaints including history of cancer; poor oral hygiene; high carbohydrate intake, high fructose diet, and a low-fat diet; previous history of tick bites; low vitamin D status; rheumatoid arthritis (RA), severe chronic back and joint pain, and type 2 diabetes. He refused pharmaceutical drugs per his choice. In addition, he almost never participated in traditional healthcare visits. He decided to participate in the HRP but was considering Adalimumab because of constant pain, pending the outcome of the HRP. The health revival process included supplements to match identified deficiencies and semi-monthly 45-minute coaching sessions.

**Figure 8:**
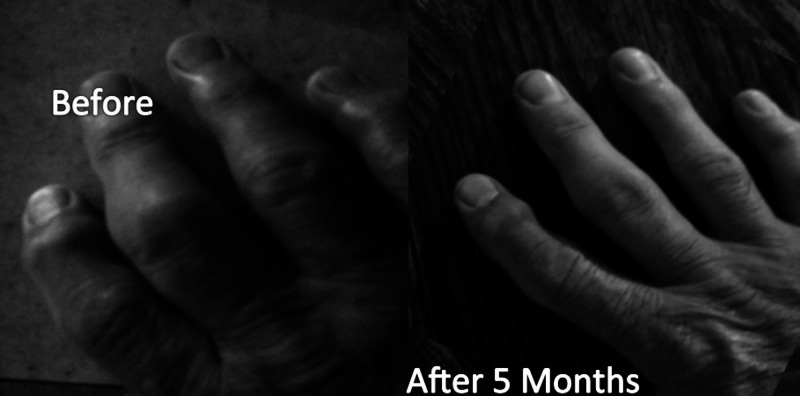
Visual change in severe rheumatoid arthritis after five months in the health revival program (HRP)

Over the first five months, the participant lost 25 pounds through a reduction in carbohydrate consumption, but with no significant change in daily calorie intake. The participant embarked on a substitution diet where, over five months, gluten-containing foods were removed from his diet and replaced with vegetables and marine- and animal-based fats. He was also put on a modest supplementation program including cod liver oil (10 g/day); vitamin D3 (5000 IU/day); magnesium glycinate (400 mg/day); vitamin K2 (50 mcg/day) and a multivitamin/mineral (per label instruction). His type 2 diabetes was reversed as illustrated by his A1C dropping from 8.8% to 5.4% and his fasting glucose dropping from 180 to < 90 mg/dL. His pain was substantially eliminated, based on a subjective pain score of 8/10 initially, to 0/10. His RA improved to enable him to be able to bend his fingers into a full fist for the first time in over five years (Figure 8).

Case 3: Polychondritis, breast cancer, cataracts - 42-year-old female factory worker with high school education (Table 15).

**Table 15:** Case 3: CDA grade and CDT before and after six months in the health revival program (HRP)

Marker	Before	After
CDA	C^-^	C^+^
CDT	104.1	101.5

This participant reported with a severe autoimmune disease, polychondritis, that produced monthly painful flares in cartilage above her shoulders including her ears and eyes. Long-term use of steroidal anti-inflammatories were implicated in the cataracts and a breast lump that was removed surgically. The cataracts had progressed sufficiently to cause her to be on disability and be unable to drive a car. Cataract surgery was not an option due to the severity and unpredictability of eye flares that could cause extremely adverse outcomes if they coincided with surgery. She had seen several specialists including local rheumatologists, natural doctors, and doctors from Cleveland Clinic with no relief to her condition. She had researched polychondritis on her own, prior to joining this program and eliminated gluten and dairy from her diet but this change did not alter the disease severity or frequency.

This participant had made significant changes in her lifestyle prior to this program as reflected in her CDA grade, but these changes were insufficient to improve her blood biomarkers indicated by the high CDT value of 104.1, indicative of serious health risk and poor prognosis. Our health revival process guided her to continued better choices and involved semi-monthly 45-minute lifestyle coaching. The main changes made over a six-month period included: increasing healthy fats, reducing carbohydrate intake, increasing micronutrient density, stopping nicotine dependence, improving digestive health with optimizing food choices including increasing stomach acid status, and repopulating gut microflora. At month 6 in the program, her eye and ear flares had subsided sufficiently to allow for a meaningful reduction in eye and oral steroids, (50 mg/day to 5 mg/day prednisone). In addition, she was able to have successful cataract surgery which enable her to start driving again, and return to work, both of which were curtailed over one year. The polychondritis may never be cured, however, with appropriate lifestyle management, it is no longer impacting her quality of life.

## Discussion

Prevention and reversal of chronic and non-communicable diseases continue to be a largely unmet need. A fresh approach is clearly warranted to curb this global scourge. One impediment is the lack of precision and personalization of risk with “poor diet” as an example. And there is a lack of measurement of a broad array of minor, yet important, risks that can easily be overcome. The same suite of risks is continually presented to individuals who historically have not been able to modify or overcome them, with smoking or alcohol consumption as examples. According to Khullar in, "We’re Bad at Evaluating Risk, How Doctors Can Help,” a broader approach involves helping patients systematically identify what’s important to them, and based on these goals and preferences, suggesting to them how to think about their options [29]. This logic is best applied across the entire time-line that defines the slow and insidious development of chronic disease. It starts with lifestyle decisions and habit development early in life that perpetuate into mid-life and then into old age. Measurement and a proper medical “workup” regardless of presumed health status is a key strategy and potential motivating factor that is currently lacking.

Changes in chronic disease biomarkers in asymptomatic people may afford an early warning sign of stealth changes to which many may respond. Pathology changes, identified with advanced diagnostics, which generally develop after a long incubation period detectable with proper biomarker evaluation, may facilitate change in the more recalcitrant. Each individual has their own motivations. Thus, providing patients with an array of choices and recommendations along the health/disease continuum has a higher probability of inciting action and improving outcomes or preventative actions.

This study evaluated a new population risk and health assessment and mitigation system where measurements of risk and disease were made across the disease continuum by using finely tuned biomarkers and risk assessment. The output was a broad-reaching care plan assembled through integration of current health survey results, biomarkers, problems, complaints, medications, vital signs, verbal input from the participant to the health coach, and contributions from the care team. The remediation path to improved health developed as a consensus between the participant and care team, of agreed upon steps and actions, that were malleable as the process moved forward. Adjustments were made based on participant preferences, success and failures, a solid health data. According to Khullar, “Patients need to understand their values but also their possible futures. The idea is not to reduce uncertainty, but to help patients clearly envision what life would look like in one outcome versus another, and to better prepare them for the various futures that might unfold.” This program was designed to give participants options beyond the management of disease once it has struck. And it included regular monitoring and concomitant course adjustments to help participants attain their goals.

This study prospectively observed adults with chronic conditions and unresolved health complaints that remained unresolved under usual care management and treatment. Following six months of HRP, participants achieved subjective and objective improvement in health status with 90% seeing a reduction in multiple blood-based biomarkers and 94% achieving a reduction is a broad measure of determinants of health risk factors. Concurrently participants reported weight loss (34% of the total and 80% of those with a reported weight loss goal), reduction in reported pain, sleeplessness, memory issues, heartburn, skin rashes, migraines, and daily fatigue. The diabetics in the program had all progressively worsen over the previous two years, as measured by fasting glucose, HbA1C, and medication usage and all improved under the HRP program.

HRP meaningfully improved HbA1c, fasting insulin, neutrophil-to-lymphocyte ratio, hs-CRP, vitamin D, white blood cell counts, red blood cell distribution width, absolute neutrophils - all part of the CDT panel. In addition, HDL, fasting glucose, triglycerides, GFR, Atherogenic Index of Plasma (AIP) liver enzymes, and blood pressure improved is most participants with initial abnormal values. AIP is emerging as a valuable representation of increased mortality risk. Improvement in this lab value ratio was consistent with previous studies using carbohydrate-restricted interventions. However, although the HRP included some level of carbohydrate restriction, this was not a mandate and carbohydrate consumption goals were not set. Instead, participants were afforded broader options that met each at their level of readiness to change and did not overwhelm anyone with unachievable objectives. In general, small swap-out suggestions were agreed upon at each encounter.

The “PURE” study reports are a set of studies that describe components of the nutritional approach used in the HRP. In "Fruit, vegetable, and legume intake, and cardiovascular disease and deaths in 18 countries (PURE): a prospective cohort study,” fruit, vegetable and legume consumption recommendations were 375-500 g/day to achieve maximum benefit at reducing non-cardiovascular and total mortality [30]. The HRP coaches encouraged consumption of three to four servings of these foods per day, focusing on lowest glycemic index choices. In “Associations of fats and carbohydrate intake with cardiovascular disease and mortality in 18 countries from five continents (PURE): a prospective cohort study,” healthy fats were found to be indicated for a reduction of total mortality risk and saturated fats were shown to be inversely associated to stroke risk. The HRP coaches guided participants to swap out carbs, sugars, and some protein in favor of healthy fats in foods and cooking oils with emphasis on increasing saturated, mono-saturated, and marine fats. Obtaining nutritional ketosis for a two to three months window was suggested for all the diagnosed type 2 diabetics; however, none achieved sustained ketosis but their metabolic markers indicated improvement of their diabetic conditions during the 6-month HRP. This suggested that the broader, more personalized risk reduction approach of this HRP, compared to strict carbohydrate restriction, affords metabolic profile results without the potential risks associated with carbohydrate starvation in insulin-resistant subjects.

Reducing whole body inflammation was the primary objective of each encounter, not just reducing the glycemic value of food. Examples included switching out proinflammatory for anti-inflammatory cooking oils, lowering glycemic value and load of substituted foods, reducing frequency of fast food consumption, improving oral hygiene, managing stress, establishing better sleep and rest patterns, enhancing hydration, improving micronutrient density of foods consumed, establishing more frequent movement routines, and consuming more gut-supporting foods.

The regular health coach encounters that included reviewing risk factors, vitals, and medication usage, with doctor supervision, may have provided behavior reinforcement. Further, it is plausible that this multi-risk amelioration care model allowed from both broader and greater adoption and improvements compared to interventions focused on fewer factors. In this HRP we effectively leveraged credible measurement and evaluation, linked these findings to participant’s unresolved and nagging health complaints, and facilitated behavioral change leading to health improvement in most participants. The program did not rely only on usual care measures of health. Participants were not confronted with high hurdles to health improvement that often discourage engagement. Instead, the program centered around meeting a person at their level of readiness and capitalizing on small triumphs that eventually led to measurable health improvements recognized by the individual that led to a cycle of improvement rather than deterioration.

No episodes of adverse events were attributable to the HRP. One insulin-dependent type 2 diabetic participant showed a sudden increase in fasting insulin, from 1.8 to 53 μU/ml, which was reported to his PCP for medication adjustment. Several participants reported dizziness and either the HRP or PCP lowered their blood pressure medication dose that, in all cases, resolved the complaint.

Prior studies have demonstrated favorable cost reductions in broad-based wellness and disease management programs. Most of the cost-saving and health maintenance were attributed to the management of existing disease rather than prevention and required a strong evidence-based approach. A strength of this HRP was an emphasis on root-causes of and reversal of disease rather than just case management. Additionally, this study reflected a real-world workplace environment with a distribution of both white- and blue-collar workers participating and with a range of diseases and aliments. Weaknesses included a lack of a representative control group, single location and participants were mostly Caucasian. The study was not of sufficient size and duration to measure hard endpoints including mortality and adverse health events. Future studies of this nature could include multisite randomized controlled trials with greater racial and ethnic diversity, and longer duration.

## Conclusions

This highly personalized and scalable health revival study protocol demonstrated that a broad array of chronic health complaints and problems can be controlled and reversed by methodically eliminating seemingly small lifestyle-induced health risks. It also demonstrated that the lifestyle risk tool, the Chronic Disease Assessment™, and the biomarker panel, the Chronic Disease Temperature™, that were used to develop care plans, changed in correspondence with participant- and medical staff-reported health improvements. Therefore, these tools may be valuable for the measurement and mitigation of chronic disease risk and chronic diseases generally. Importantly, the implementation of this program is low cost, using inexpensive on-line survey tools, biomarkers, and health coaching. Additionally, this program is well suited to be implemented in large populations through surveying, obtaining labs through national networks, and performing group coaching sessions based on common risks identified through the risk assessment tool. More studies using this overall HRP approach are required to validate the measurement methods, processes, and outcomes. This approach offers a potentially important health delivery modality in a world with escalating chronic disease morbidity and mortality.

## Figures and Tables

**Figure 9:**
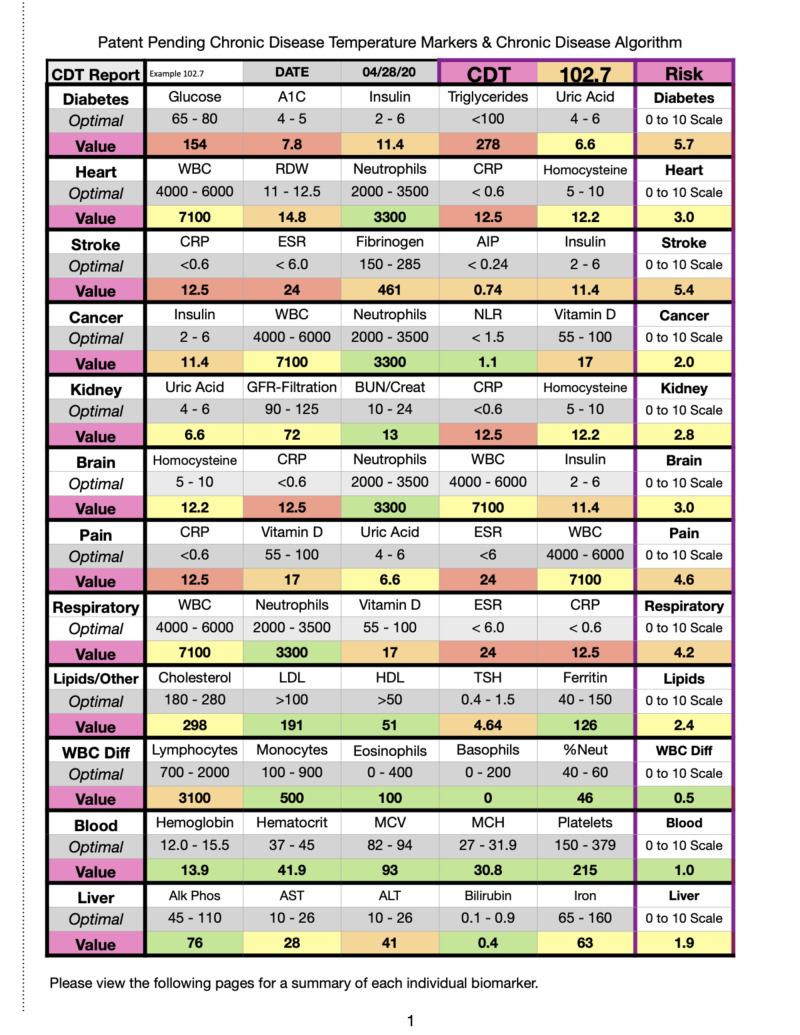
Example of a Chronic Disease Temperature Report - Medium Risk - CDT Score: 102.7 - Page 1 only

**Figure 10:**
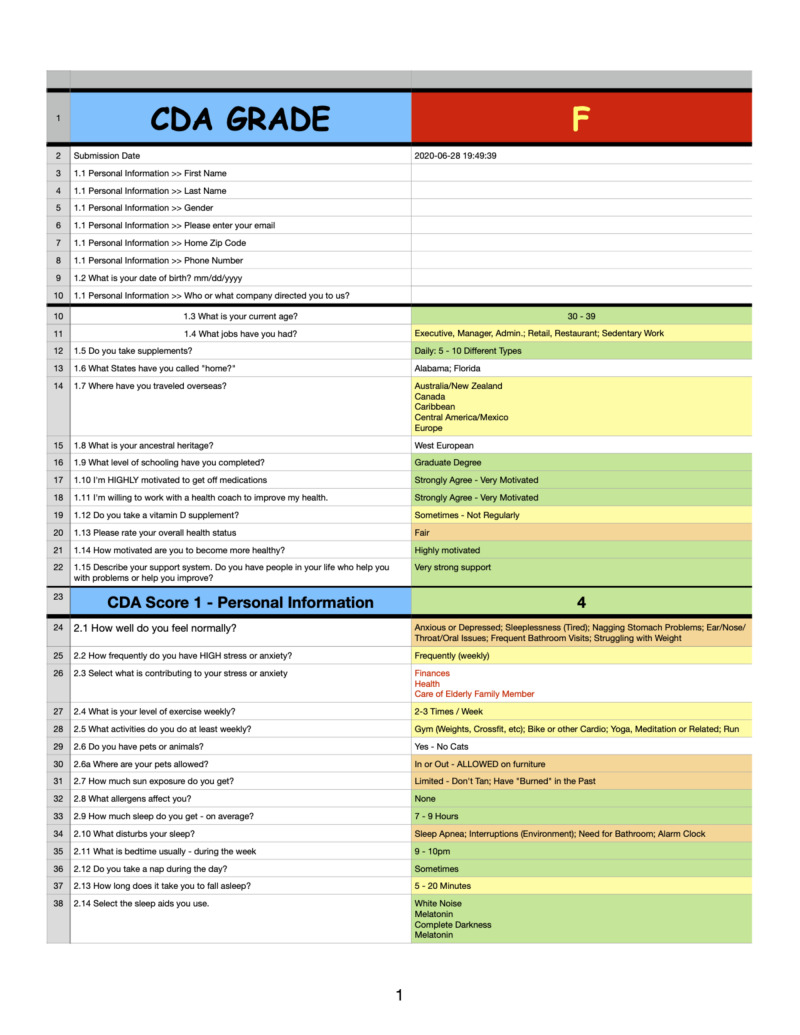
Chronic Disease Assessment color-coded report: Key: Green – low risk; yellow – mild risk; orange – moderate risk; red – severe risk; bright red – extreme risk, Page 1 of 5 only

## References

[1] World Health Organization (2020). World Health Organization. (2014). Global status report on noncommunicable diseases. diseases.

[2] Woolf SH, Aron LY (2020). The US health disadvantage relative to other high-income countries: findings from a National Research Council/ Institute of Medicine report. JAMA.

[3] McKay B, Overberg P (2020). Heart disease strikes back across the U.S even in healthy places. Wall Street J.

[4] Buttorff C, Ruder T, Bauman M (2020). Multiple Chronic Conditions in the United States. https://www.rand.org/content/dam/rand/pubs/tools/TL200/TL221/RAND_TL221.pdf.

[5] Johnson WC, Brennan N, Rodriguez S (2018). Consistently high turnover in the group of top health care spenders. NEJM Catalyst.

[6] Lewis TJ, Austin T, Carter ML (2020). The cytokine storm and pre-cytokine storm status in COVID-19 - a model for managing population risk for pandemics and chronic diseases. Emerg Infect Dis Diag J.

[7] Liu Y, Sun W, Guo Y (2020). Association between platelet parameters and mortality in coronavirus disease 2019: retrospective cohort study. Platelets.

[8] Willett WC, Koplan JP, Nugent R (2020). Prevention of chronic disease by means of diet and lifestyle changes. Disease Control Priorities in Developing Countries. 2nd edition. The International Bank for Reconstruction and Development/The World Bank.

[9] Gakidou E, Afshin A, Abajobir AA (2017). Global, regional, and national comparative risk assessment of 84 behavioural, environmental and occupational, and metabolic risks or clusters of risks, 1990-2016: a systematic analysis for the Global Burden of Disease Study 2016. Lancet.

[10] Cho YI, Lee SD, Arozullah AM (2008). Effects of health literacy on health status and health service utilization amongst the elderly. Social Sci Med.

[11] Vernon JA, Trujillo A, Rosenbaum SJ (2020). Low health literacy: Implications for national health policy. Himmelfarb Health Sci.

[12] Remmers C, Hibbard J, Mosen DM (2009). Is patient activation associated with future health outcomes and healthcare utilization among patients with diabetes?. J Ambul Care Manage.

[13] Sachdeva A, Cannon CP, Deedwania PC (2009). Lipid levels in patients hospitalized with coronary artery disease: an analysis of 136,905 hospitalizations in Get With The Guidelines. Am Heart J.

[14] De Ruijter W, Westendorp RG, Assendelft WJ (2009). Use of Framingham risk score and new biomarkers to predict cardiovascular mortality in older people: population based observational cohort study. BMJ.

[15] McEwen BS, Stellar E (1993). Stress and the individual: mechanisms leading to disease. Arch Internal Med.

[16] Kirkwood TL, Kowald A (1997). Network theory of aging. Exp Gerontol.

[17] Gay JL, Salinas JJ, Buchner DM (2015). Meeting physical activity guidelines is associated with lower allostatic load and inflammation in Mexican Americans. J Immigrant Minority Health.

[18] Wang TJ, Gona P, Larson MG (2006). Multiple biomarkers for the prediction of first major cardiovascular events and death. N Engl J Med.

[19] Edwards MK, Blaha MJ, Loprinzi PD (2017). Atherogenic index of plasma and triglyceride/high-density lipoprotein cholesterol ratio predict mortality risk better than individual cholesterol risk factors, among an older adult population. Mayo Clinic Proc.

[20] Lewis TJ, Trempe CL (2016). Quarterback Your Own Health - How to Take and Lower Your Chronic Disease Temperature. https://bit.ly/2PqMs0w.

[21] Gabay C, Kushner I (1999). Acute-phase proteins and other systemic responses to inflammation. N Engl J Med.

[22] Yu E, Rimm E, Qi L (2016). Diet, lifestyle, biomarkers, genetic factors, and risk of cardiovascular disease in the Nurses’ Health Studies. Am J Public Health.

[23] Bonds DE, Miller ME, Bergenstal RM (2010). The association between symptomatic, severe hypoglycaemia and mortality in type 2 diabetes: retrospective epidemiological analysis of the ACCORD study. BMJ.

[24] Perseghin G., Calori G, Lattuada G (2012). Insulin resistance/hyperinsulinemia and cancer mortality: the Cremona study at the 15th year of follow-up. Acta Diabetol.

[25] Ridker PM (2003). Rosuvastatin in the primary prevention of cardiovascular disease among patients with low levels of low-density lipoprotein cholesterol and elevated high-sensitivity C-reactive protein: rationale and design of the JUPITER trial. Circulation.

[26] Margolis KL, Manson JE, Greenland P (2005). Leukocyte count as a predictor of cardiovascular events and mortality in postmenopausal women: the Women’s Health Initiative Observational Study. Arch Internal Med.

[27] Giovannucci E, Liu Y, Rimm EB (2006). Prospective study of predictors of vitamin D status and cancer incidence and mortality in men. J Natl Cancer Inst.

[28] Erturk M, Cakmak HA, Surgit O (2014). The predictive value of elevated neutrophil to lymphocyte ratio for long-term cardiovascular mortality in peripheral arterial occlusive disease. J Cardiol.

[29] Khullar D. (2018). We're Bad at Evaluating Risk. How Doctors Can Help. The New York Times.

[30] Miller V, Mente A, Dehghan M (2017). Fruit, vegetable, and legume intake, and cardiovascular disease and deaths in 18 countries (PURE): a prospective cohort study. Lancet.

